# Human Salivary Histatin-1 Is More Efficacious in Promoting Acute Skin Wound Healing Than Acellular Dermal Matrix Paste

**DOI:** 10.3389/fbioe.2020.00999

**Published:** 2020-08-19

**Authors:** Xiaoxuan Lei, Liuhanghang Cheng, Haiyan Lin, Mengru Pang, Zexin Yao, Caihong Chen, Tymour Forouzanfar, Floris J. Bikker, Gang Wu, Biao Cheng

**Affiliations:** ^1^Department of Oral and Maxillofacial Surgery/Pathology, Amsterdam UMC and Academic Center for Dentistry Amsterdam (ACTA), Vrije Universiteit Amsterdam (VU), Amsterdam Movement Science, Amsterdam, Netherlands; ^2^Department of Burn and Plastic Surgery, General Hospital of Southern Theater Command, Guangzhou, China; ^3^Savaid Stomatology School, Hangzhou Medical College, Hangzhou, Zhejiang, China; ^4^Department of Oral Biochemistry, Academic Center for Dentistry Amsterdam (ACTA), University of Amsterdam (UvA) and Vrije Universiteit Amsterdam (VU), Amsterdam, Netherlands; ^5^Department of Oral Implantology and Prosthetic Dentistry, Academic Center for Dentistry Amsterdam (ACTA), University of Amsterdam (UvA) and Vrije Universiteit Amsterdam (VU), Amsterdam, Netherlands

**Keywords:** Histatin1, acellular dermal matrix, acute wound, inflammatory response, angiogenesis, animal model

## Abstract

A rapid wound healing is beneficial for not only recovering esthetics but also reducing pain, complications and healthcare burdens. For such a purpose, continuous efforts have been taken to develop viable dressing material. Acellular dermal matrix (ADM) paste has been used to repair burn wounds and is shown to promote angiogenesis as well as fibroblast attachment and migration. However, its efficacy still needs to be significantly improved to meet clinical demands for accelerating acute skin wound healing. To approach this problem, we studied the added value of a human salivary peptide — Histatin 1 (Hst1). Hst1 was chosen because of its potency to promote the adhesion, spreading, migration, metabolic activity and cell-cell junction of major skin cells and endothelial cells. In this study, we hypothesized that ADM paste and Hst1 showed a better effect on the healing of surgically created acute skin wounds in mice since ADM paste may act as a slow release system for Hst1. Our results showed that the healing efficacy of 10 μM topically administrated Hst1 was significantly higher compared to the control (no Hst1, no ADM) from day 3 to day 10 post-surgery. In contrast, ADM alone failed in our system at all time points. Also, the combination of ADM paste and Hst1 did not show a better effect on percentage of wound healing. Histological analysis showed that 10 μM Hst1 was associated with maximal thickness of newly formed epidermal layer on day 7 as well as the largest collagen area on day 14. In addition, immunohistochemical staining showed that the number of CD31-positive blood vessels in the group of 10 μM Hst1 was 2.3 times compared to the control. The vascular endothelial growth factor (VEGF) expression in the groups of 10 μM Hst1 group and ADM + 10 μM Hst1 group was significantly higher compared with the control group. Furthermore, 10 μM Hst1 group was associated with significantly lower levels of CD68-positive macrophage number, interleukin-1β (IL-1β) expression and C-reactive protein (CRP) expression than those of the other groups (control, ADM alone and ADM + 10 μM Hst1). In contrast, ADM was only associated with significantly lower CD68-positive macrophage number and IL-1β expression in comparison with the control. The co-administration of Hst1 and ADM paste did not yield more beneficial effects than Hst1 alone. In conclusion, the topically administrated of 10 μM Hst1 could be a promising alternative dressing in managing acute wound healing.

## Introduction

Acute skin wounds may be caused by trauma, burns, lacerations or abrasions and surgery. There is an increasing demand in clinic to accelerate wound healing in order to rapidly recover esthetics as well as reduce pain, potential complications and healthcare burdens (Sen et al., 2009). In general terms, the process of wound healing can be divided into four consecutive phases: vascular response, inflammatory response, proliferation and maturation (Flanagan, 2000). Right after acute injury, exposure of wounds to air initiates clotting processes that are mediated by platelet aggregation and coagulation cascades. In turn, the activation of clotting factors stimulates the release of pro-inflammatory mediators, such as TNF-α (tumor necrosis factor-α) and IL-1β (interleukin-1β) so as to recruit inflammatory cells, such as neutrophils and macrophages (Naldini and Carraro, 2005). Macrophages can display two different functional phenotypes, such as M1 (classically activated) and M2 macrophages (alternatively activated) (Hubner et al., 1996; Werner and Grose, 2003), both of which play important roles in wound healing process in different ways. As early as day 1 after injury, M1 macrophages infiltrate into wounds areas and direct host-defense against pathogens through phagocytosis and secreting pro-inflammatory cytokines such as TNF-α, IL-1β, IL-6 (interleukin-6) (Hesketh et al., 2017), while M2 macrophages are responsible to pacify inflammation and activate tissue repair. At 48 and 72 h after injury, M1 macrophages transit to M2 macrophages and secrete a series of growth factors including TGF-β1 (transfer growth factor-β1) and VEGF-α (vascular endothelial growth factor-α), which promote angiogenesis and re-epithelialization (Vannella and Wynn, 2017; Klar et al., 2018). These growth factors also stimulate the synthesis and secretion of large amounts of collagen fibers and matrix components by fibroblasts to form connective tissues (Liebl and Kloth, 2012). Continuous attempts have been made to develop suitable wound dressings to accelerate wound healing by targeting and stimulating the abovementioned biological events.

One viable would dressing material in clinic is acellular dermal matrix (ADM) (Patton Berry and Kralovich, 2007; Vardanian et al., 2011). ADM is a cell-free extracellular matrix (ECM), containing collagen bundles, elastic fibers, proteoglycan and glycosaminoglycan (Walter et al., 1998). ADM has good histocompatibility and low antigenicity. ADM is shown to accelerate wound healing by promoting the adhesion, migration, proliferation, metabolism, differentiation and survival of major skin cells (Ghatak et al., 2015) through its various components. For example, glycosaminoglycan peptide released from ADM can inhibit the recruitment of neutrophils to the site of inflammation and promote angiogenesis by stimulating nitric oxide production (Peplow, 2005; Taylor and Gallo, 2006). ADM also contains a large amount of RGD peptide (Arg-Gly-Asp) (Walter et al., 1998), which can promote the migration of fibroblasts through binding integrin on cell membrane and activating cell-substrate interaction (Ghosh et al., 2006). However, its efficacy still needs to be significantly improved to meet clinical demands for accelerating acute skin wound healing. Consequently, in order to increase treatment of success, it is of utmost importance to seek for novel bioactive agents that promote wound healing.

Histatin 1 (Hst1) is a major member of higher primates’ saliva-derived histidine-rich peptide family and shows a promising application potential to promote skin wound healing (Oudhoff et al., 2009a). Hst1 has been shown to promote the adhesion, spreading, migration and cell-cell junction of epithelial cells (Oudhoff et al., 2008; van Dijk et al., 2015; van Dijk et al., 2017) on both bioactive (van Dijk et al., 2015) and bio-inert surfaces (van Dijk et al., 2017). Comparable effects are found on endothelial cells and fibroblasts (van Dijk et al., 2015; Torres et al., 2017). Our recent study shows that the underlying mechanisms for Hst1’s effects are highly different from ADM: Hst1 directly activates cell functions through its subcellular targets (Ma et al., 2020). The promoting effects of Hst1 on angiogenesis seem to be mediated by the Ras and Rab interactor 2 (RIN2)/Rab5/Rac1 signaling axis (Torres et al., 2017). A recent *in vivo* study corroborates that Hst1 can promote skin wound healing by promoting cell migration and angiogenesis (Lin et al., 2020). However, Hst1 may be easily degraded by enzymes when applied to wound surface (McDonald et al., 2011). These characteristics inspired us to co-administrate ADM paste and Hst1 since ADM paste might provide a slow delivery system so as to potentiate the wound-healing efficacy of Hst1. Furthermore, ADM and Hst1 take effects via different signaling pathways, which may compensate each other, yielding a better effect. We hypothesized that the co-administrated ADM paste and Hst1 might yield a better effect on the healing of acute wounds in mice since ADM paste might provide a slow release system for Hst1.

In this study, we explored the effects of Hst1 (at different concentrations), ADM paste and their combination on full-thickness skin defects in mice to evaluate wound healing rate, re-epithelialization, collagen expression, angiogenesis and inflammatory response.

## Materials and Methods

### Hst1/ADM Preparation

The Hst1 (≥95% purity) was synthesized and supplied by the manufacturer (SynPeptide Co., Ltd., Nanjing, China). Hst1 was dissolved in 1 ml 0.9% NaCl and supplemented with Hst1 formulations at final concentrations of 1, 10, and 50 μM. The ADM (Jiangsu Unitrump Biomedical Technology Co., Ltd., Nanjing, China) is an enzymatically digested product in a form of ultrafine powder with an average particle size at 33.4 μm (Liu and Sun, 2020). Before application, ADM powder was mixed with different concentrations of Hst1 at a volume ratio of 1:1 (v/v) through three-way pipes for 1 h, forming into pastes. To ensure consistency of treatment, we mixed granular ADM with 0.9% NaCl to obtain ADM paste. The treatments were divided into eight groups as follows:

• G1: no Hst1, no ADM;
• G2: ADM alone;
• G3: 1 μM Hst1 alone;
• G4: 10 μM Hst1 alone;
• G5: 50 μM Hst1 alone;
• G6: ADM + 1 μM Hst1;
• G7: ADM + 10 μM Hst1;
• G8: ADM + 50 μM Hst1.

### Acute Wound Model *in vivo*

A total of 48 male C57 mice, aging 6–8 weeks, weighing 22–28 g was purchased from the Animal Research Center of Guangdong (Guangzhou, China). The usage of these animals was approved by the Animal Care Committee of General Hospital of Southern Theater Command. The animal ethics approval number was 2019051701.

Briefly, dorsal hair of the mice was carefully shaved-off and sterilized with iodophor. Animal anesthesia was achieved with an intraperitoneal injection of 1% pentobarbital sodium (5 ml/kg). After anesthesia, a rounded, full-thickness, 1 cm acute wound was made by an impression with a punch biopsy instrument in the dorsal area of each mouse, followed by excising of the full-thickness skin. Eight different treatments were assigned to the wounds randomly, and each wound was treated with a 0.5 ml dose every other day until complete healing. The wounds were photographed on days 0, 3, 5, 7, 10, 14 post-surgery. On days 7 and 14, three mice were randomly sacrificed from each group at each time point and took wound tissues to do the detection.

### Wound Healing Rate

On days 0, 3, 5, 7, 10, and 14 post-surgery, the wounds were photographed with a Nikon digital camera and the wound area measured by Image J software. A1 represented the original wound area. AX represented the wound area at each time point post-surgery. The percentage of wound healing over time was calculated using the formula: percentage of wound healing (%) = [(A1 - AX)/A1] × 100%.

### Hematoxylin and Eosin (H&E) Staining

The wound tissues were fixed in 4% paraformaldehyde for 48 h, dehydrated with gradient alcohol and subsequently embedded in paraffin with an embedding machine (JB-P5; Junjie Electronics Inc., Wuhan, China) and sliced in a thickness of 4 μm, and dried in an oven (Shanghai Huitai Instrument Manufacturing Co., Ltd., Shanghai, China) at 65 for 2 h. The sections were dehydrated and stained with hematoxylin and eosin and photographed by an electron microscope (Olympus BX51, Hamburg, Germany). Five tissue sections in each group were selected, with the area and length of re-epithelium measured by Image J software. The epithelial area divided by the epithelial length equaled the epithelial thickness.

### Masson Staining

To assess collagen production in all groups, Masson staining was carried out. The tissue sections were deparaffinized and dehydrated and stained using hematoxylin (G1004; ServiceBio Inc., Boston, MA, United States), differentiated with hydrochloric acid-alcohol for 5 s and stained with a ponceau-acid fuchsin (G2011; ServiceBio Inc., Boston, MA, United States) for 5 min. The stained sections were placed in 1% phosphomolybdic acid for 2 min and stained with aniline blue (G1071; ServiceBio Inc., Boston, MA, United States) for 5 min and subsequently dehydrated and sealed with neutral gum (G1403; ServiceBio Inc., Boston, MA, United States). The stained sections were photographed by the microscope and the collagen expression level was analyzed using Image-Pro Plus software.

### Immunohistochemistry

To evaluate angiogenesis and inflammatory factors in all groups, immunohistochemistry staining was performed. Sections were deparaffinized and blocked with 3% bovine serum albumin (BSA). Subsequently, sections were incubated overnight at 4°C with antibodies rabbit-anti platelet endothelial cell adhesion molecule-1 (CD31) (GB13063; 1:300; Servicebio Inc., Boston, MA, United States), mouse-anti VEGF (MA5-13182; 1:100 Thermo Fisher Scientific Co., Ltd., CA, United States), hamster-anti cluster of differentiation 68 (CD68) (GTX80302; 1:500; Genetax), rabbit-anti TNF-α (ab6671; 1:100; Abcam Trade Co., Ltd., Shanghai, China), rabbit-anti C-reactive protein (CRP) (bs-0155R; 1:200; Bioss Biotechnology Co., Ltd., Beijing, China), rabbit-anti IL-6 (bs-0782; 1:200; Bioss Biotechnology Co., Ltd., Beijing, China) and rabbit-anti IL-1β (ab9722; 1:100; Abcam Trade Co., Ltd., Shanghai, China). Correspondingly, the sections were incubated with secondary goat anti-mouse (G1214; 1:200; ServiceBio Inc., Boston, MA, United States) or goat anti-rabbit (G1213; 1:200; ServiceBio Inc., Boston, MA, United States) antibodies at 37°C for 50 min, and developed with h 3,3′-diaminobenzidine tetrahydrochloride (DAB) solution (G1211; ServiceBio Inc., Boston, MA, United States) for 10 min and counterstained the nuclei with hematoxylin. The stained sections were dehydrated and sealed with neutral gum, and photographed by microscope and analyzed the positive expression of the observations by Image-Pro Plus. Five tissue sections in each group were selected, with the area and integrated option density (IOD) measured by Image-Pro Plus. The positive expression level of VEGF, CRP, IL-1β, IL-6 and TNF-α was calculated using the formula: Mean Optical Density (MOD) = IOD Sum/Area Sum. Image-pro plus was chosen for the analysis of CD31-positive blood vessels. Images from five tissue sections in each group and five different fields of each section were submitted to the software for analysis. The colors of positive staining were recorded and counted automatically.

### Statistical Analysis

Statistical analysis was performed by SPSS software. All quantitative data were expressed as mean value ± standard deviation (SD). One-way ANOVA and Bonferroni tests were used to analyze significance levels. *P* < 0. 05 was considered statistically significant.

## Results

### The Effects of Hst1, ADM Paste and Their Combination on Wound Healing Percentage Over Time

**FIGURE 1:**
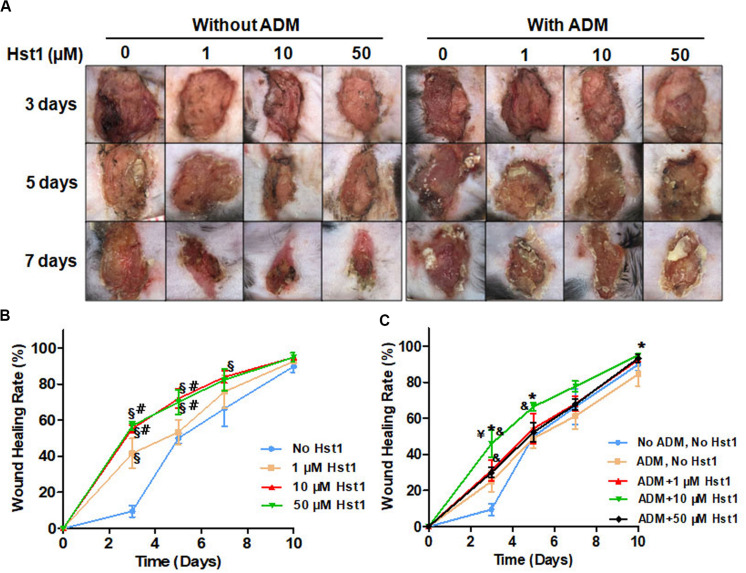
3, 5, 7, and 10 days post-surgery, the dynamic changes of wound area in different groups after treatment. **(A)** Representative images of acute wounds showed that the wound area treated by 10 μM Hst1 was significantly smaller than the other groups; **(B)** The temporal changes of wound healing percentage (%) in each treatment group. On days 3, 5, and 10, the healing percentage of the wounds treated by 10 μM Hst1 was significantly higher than the control; **(C)** ADM + 10 μM Hst1 was associated with significantly higher healing percentage than the ADM and the control on days 3 and 5. Data were presented as mean ± SD. §: the 1, 10, 50 μM Hst1 group vs the control group; #: the 10, 50 μM Hst1 group vs the 1 μM Hst1 group; &: the 1, 10, 50 μM Hst1 + ADM group vs the control group; *: the 10 μM Hst1 + ADM group vs the ADM group. –Y: the 10 μM Hst1 + ADM group vs the 50 μM Hst1 + ADM group (*P* < 0.05).

3, 5, 7, and 10 days post-surgery, the healing of the acute wounds in different groups progressed with time in different percentages (Figure 1A). On day 3, 5 and 10, the healing percentage of the wounds treated by 10 μM Hst1 was significantly higher compared to the control (no Hst1), while Hst1 at 50 μM showed such a superior efficacy only on day 3 and 5 (Figure 1B). The healing efficacies of Hst1 at 10 and 50 μM were significantly higher than Hst1 at 1 μM on day 3 and day 5. The most beneficial effect of Hst1 was detected on day 3. At this time point, the wound healing percentage in the groups of Hst1 at 50, 10, and 1 μM were 5.90, 5.77 and 4.34 times that of the control (Figure 1B). ADM alone showed only a mild, yet insignificant beneficial effect on wound healing on day 3 when compared with the control group (Figure 1C). Its effects rapidly tapered with its average percentage wound healing even lower than the control group on day 5 to day 10. ADM + 1 μM Hst1 and ADM + 50 μM Hst1 were associated with significantly higher healing percentage than the control only on day 3 (Figure 1C). ADM + 10 μM Hst1 resulted a significantly higher healing percentage than the ADM on day 3, 5 and 10. According to these results, we adopted Hst1 at its optimal concentration (10 μM) and corresponding groups, such as control (no ADM, no Hst1), ADM and ADM + 10 μM Hst1 group in the subsequent histological and immunohistochemical assessments.

### The Effects of Hst1, ADM Paste and Their Combination on Dermal Thickness and Collagen Regeneration

**FIGURE 2:**
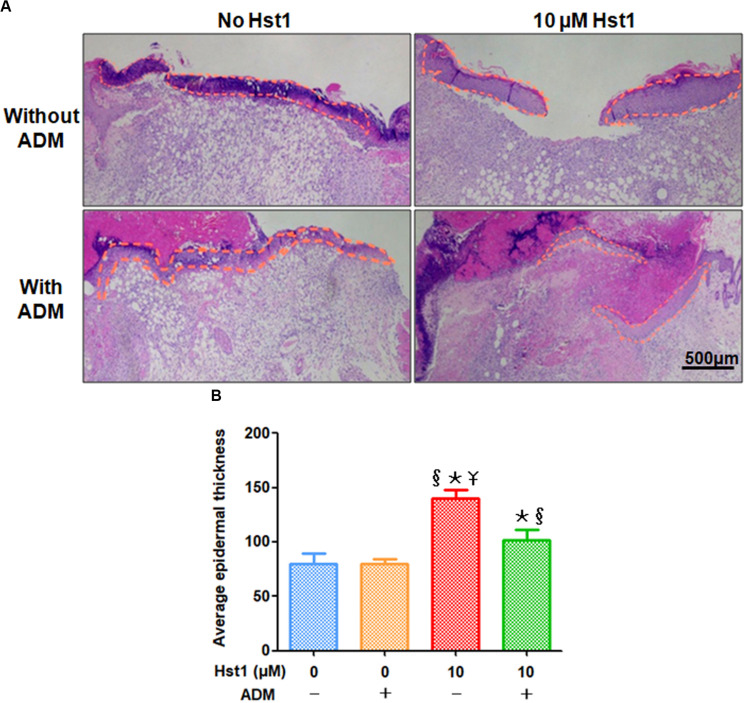
Histological evaluation of epidermal thickness at wound sites. **(A)** 7 days post-surgery, the newly formed epidermal in the 10 μM Hst1 group was thicker and cell stratification was more obvious. The yellow curve showed the epidermal regeneration area. The scale bar = 500 μm; **(B)** Quantitative analysis showed that the treatment with 10 μM Hst1 was associated with the highest thickness of newly formed epidermal layer, which was followed by ADM + 10 μM Hst1. In contrast, ADM alone didn’t promote epidermal thickness. §: The 10 μM Hst1 group and 10 μM Hst1 + ADM group vs the control group; *: The 10 μM Hst1 group and 10 μM Hst1 + ADM group vs the ADM group; –Y: The 10 μM Hst1 group vs the 10 μM Hst1 + ADM group (*P* < 0.05).

**FIGURE 3:**
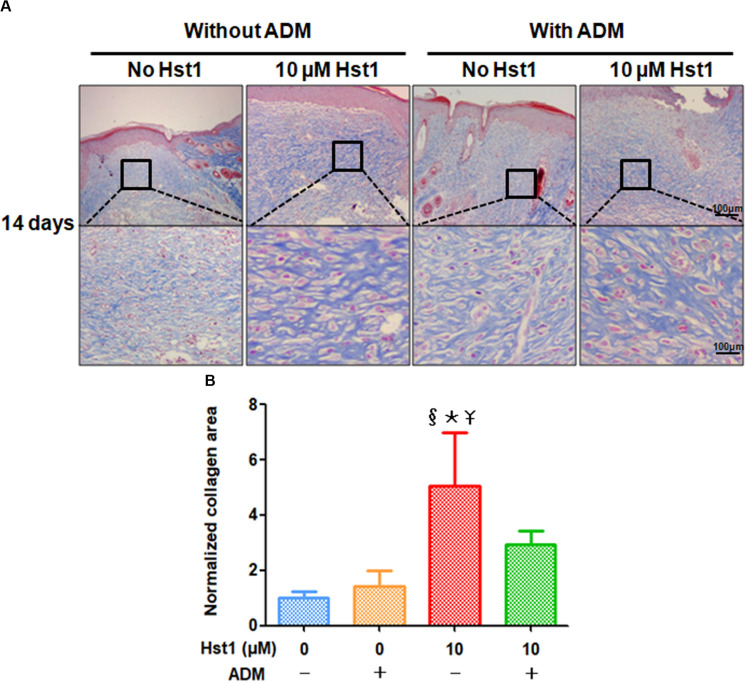
Masson staining evaluation of collagen fibers deposited in newly formed epidermal layer. **(A)** 14 days post-surgery, the collagen fibers in the 10 μM Hst1 group deposited massively and arranged regularly. Blue represented the positive expression. The scare bar = 100 μm; **(B)** Quantitative analysis by normalizing collagen area in treatment groups to the control group. The collagen regeneration area in the 10 μM Hst1 group was significantly larger compared with the other groups, while either ADM or ADM + 10 μM Hst1 didn’t significantly influence collagen area. §: The 10 μM Hst1 group vs the control group; *: The 10 μM Hst1 group vs the ADM group; –Y: The 10 μM Hst1 group vs the 10 μM Hst1 + ADM group (P < 0.05).

To investigate the effects of 10 μM Hst1 + ADM on re-epithelialization and collagen regeneration, the tissue samples were stained with HE staining (Figure 2A) and Masson staining (Figure 3A), respectively. The thickness and area of newly formed epidermis in each group were measured. On day 7 post-surgery, the newly formed epidermal in the 10 μM Hst1 group was thicker and cell stratification was more obvious (Figure 2A). Quantitative analysis showed that the treatment with 10 μM Hst1 was associated with the highest thickness of newly formed epidermal layer, which was followed by ADM + 10 μM Hst1 (Figure 2B). In contrast, ADM alone didn’t promote epidermal thickness (Figure 2B).

14 days post-surgery, the collagen area in newly formed dermal layer (Figure 3A) was measured and analyzed. The collagen regeneration area in the 10 μM Hst1 group was significantly larger compared with the other groups, while either ADM alone or ADM + 10 μM Hst1 did not significantly influence collagen area (Figure 3B).

### The Effects of Hst1, ADM Paste and Their Combination on Angiogenesis

**FIGURE 4:**
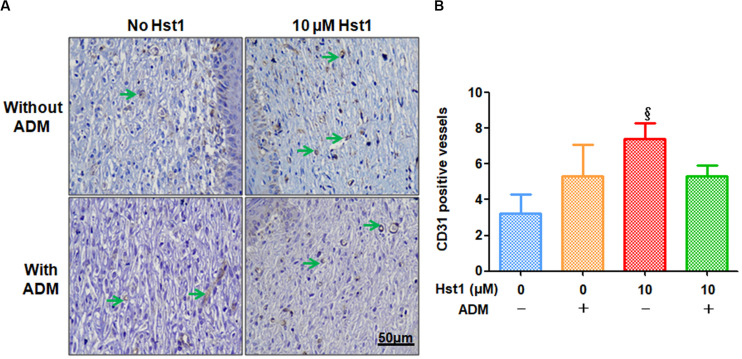
Immunohistochemical evaluation of CD31-positive blood vessels at wound sites. **(A)** 14 days post-surgery, in the CD31-positive blood vessels (in green arrows) per microscopic field (7.4 ± 0.9) in the 10 μM Hst1 group was significantly higher than the control group (3.2 ± 1.1). The scale bar = 50 μm; **(B)** Quantitative analysis of the number of CD31-positive blood vessels. The 10 μM Hst1 group presented a higher number of new blood vessels compared with the other groups. §: The 10 μM Hst1 group vs the control group (*P* < 0.05).

**FIGURE 5:**
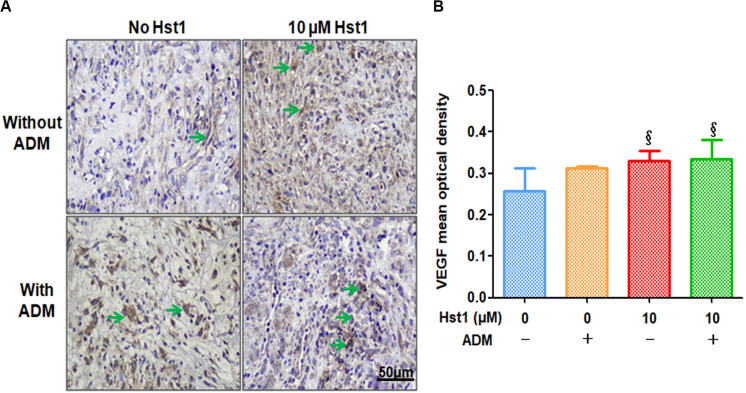
Immunohistochemical evaluation of angiogenic marker VEGF positive expression. **(A)** 14 days post-surgery, the positive expression level of VEGF (in green arrows) in the 10 μM Hst1 group was higher. The scale bar = 50 μm. **(B)** Quantitative analysis of the VEGF expression level at wound sites. 14 days post-surgery, the VEGF expression in the groups of 10 μM Hst1 and ADM + 10 μM Hst1 was significantly higher compared with the control group and ADM group. §: The 10 μM Hst1 group and 10 μM Hst1 + ADM group vs the control group (*P* < 0.05).

Angiogenic markers (CD31 and VEGF) were stained using immunohistochemistry to investigate the effects of Hst1, ADM and their combination on angiogenesis at the wound sites (Figures 4A, 5A). 14 days post-surgery, in the CD31-positive blood vessels per microscopic field in the group of 10 μM Hst1 (7.4 ± 0.9) was significantly higher than that of the control group (3.2 ± 1.1) (Figure 4B). No significant difference was found between the control group and ADM (5.3 ± 1.7) or ADM ± 10 μM Hst1 (5.3 ± 0.58) (Figure 4B). 14 days post-surgery, the VEGF expression in the groups of 10 μM Hst1 group and ADM + 10 μM Hst1 group was significantly higher compared with the control group and ADM group (Figure 5B).

### The Effects of Hst1, ADM Paste and Their Combination on Inflammation

**FIGURE 6:**
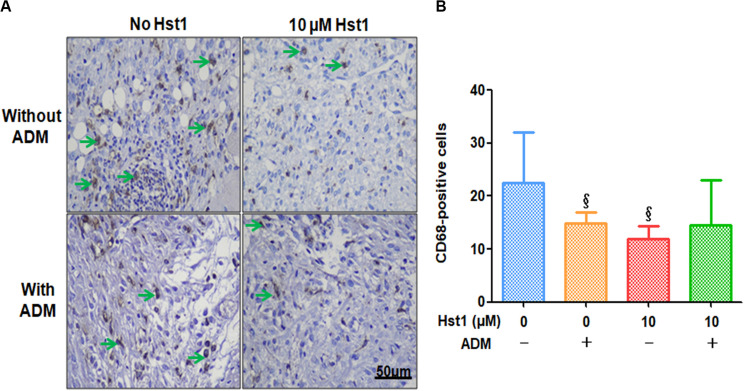
Immunohistochemical evaluation of CD68-positive cells at wound sites. **(A)** 7 days post-surgery, the number of CD68-positive macrophages (in green arrows) in the groups of ADM (14.8 ± 2.0) and 10 μM Hst1 group (12.0 ± 2.3) were significantly lower than the control group (22.6 ± 9.4). The scale bar = 50 μm; **(B)** Quantitative analysis of the number of CD68-positive cells. The 10 μM Hst1 group presented significantly lower amounts of macrophages compared with the control group. §: The 10 μM Hst1 group and ADM group vs the control group (*P* < 0.05).

To investigate the effect of ADM + 10 μM Hst1 on inflammatory response, the CD68 macrophages at the wound site were measured and analyzed (Figure 6A). 7 days post-surgery, the number of CD68-positive macrophages in the groups of ADM (14.8 ± 2.0) and 10 μM Hst1 group (12.0 ± 2.3) were significantly lower than the control group (22.6 ± 9.4) (Figure 6B). No significant difference in this parameter was found between the ADM + 10 μM Hst1 (14.4 ± 8.5) and 10 μM Hst1.

**FIGURE 7:**
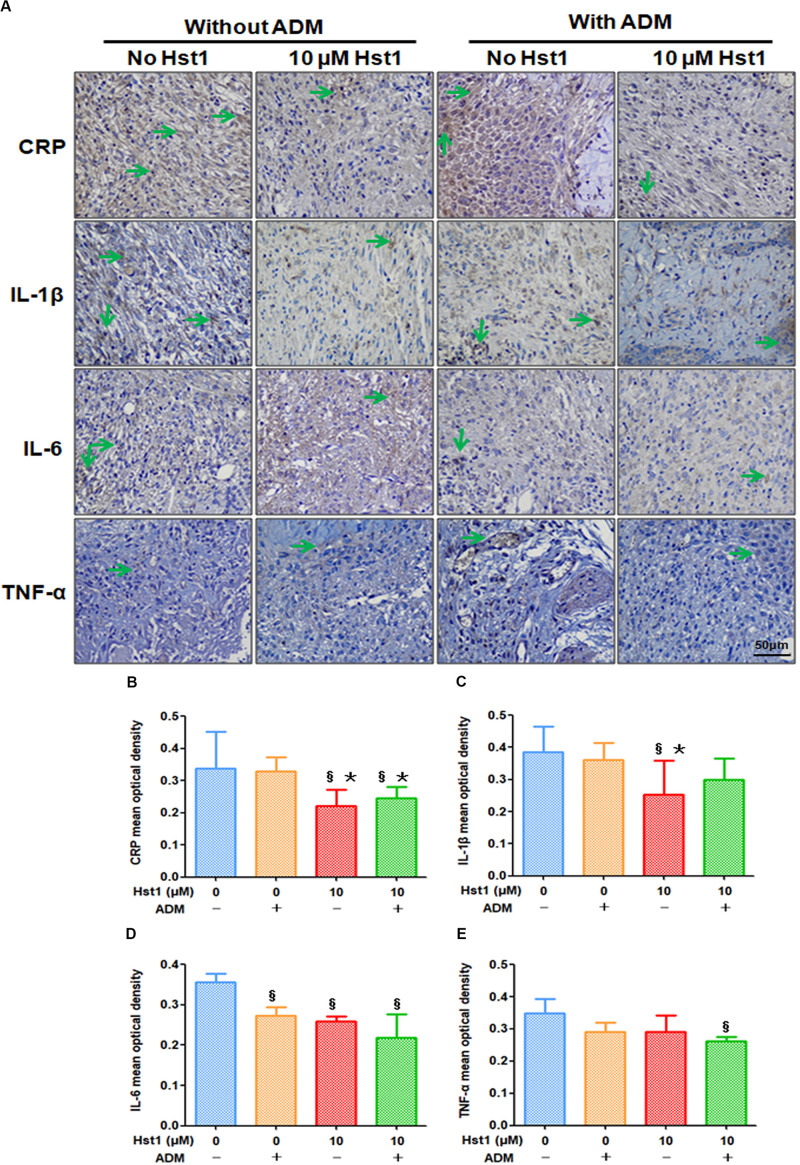
Immunohistochemical evaluation of the expression levels of CRP, IL-1β, IL-6, TNF-α at wound sites. **(A)** 14 days post-surgery, the positive expression level of CRP, IL-1β, IL-6, TNF-α (in green arrows) in the 10 μM Hst1 group was lower. The scale bar = 50 μm; **(B–E)**. Quantitative analysis of the expression level of CRP, IL-1β, IL-6, TNF-α at wound sites. On day 7 post-surgery, 10 μM Hst1 was associated with significantly lower levels of CRP, IL-1β, IL-6 and TNF-α than the control group. §: The 10 μM Hst1 group, 10 μM Hst1 + ADM group and ADM group vs the control group; *: The 10 μM Hst1 group and 10 μM Hst1 + ADM group vs the ADM group (*P* < 0.05).

The CRP, IL-1β, IL-6 and TNF-α mainly produced by fibroblasts and macrophages were conducive to inflammation, which could be used for indicating inflammatory response at the wound site (Figure 7A). On day 7 post-surgery, 10 μM Hst1 was associated with significantly lower levels of CRP, IL-1β and IL-6 than the control group (Figures 7B–D). No significant difference in any of these inflammatory parameters between 10 μM Hst1 and ADM + 10 μM Hst1 (Figures 7B–E).

## Discussion

Skin provides barrier functions against physical damage, pathogens and dehydration, contributing to the maintenance of body health and homeostasis (Nejati et al., 2013). In order to avoid undesired-injury related (side) effect, such as infection and locally poor blood supply, skin wound healing should be accelerated in order to restore its integrity and functionality (Canedo-Dorantes and Canedo-Ayala, 2019). Furthermore, rapid wound healing can also reduce the swelling and pain of wounds, thus reducing suffer of patients. In this study, we showed that the addition of 10 μM Hst1 efficaciously promoted the healing of acute wounds, while ADM alone showed only mild effects. Furthermore, the co-administration of ADM paste and 10 μM Hst1 was not superior to 10 μM Hst1. Consequently, a topical administration of 10 μM Hst1 is promising in clinic to promote the acute wound healing. To our knowledge, this is the first study to report the efficacy of topically administrated Hst1 on skin wound healing.

A viable dressing should be able to take effect rapidly so as to promote skin wound healing in early stages. ADM, a clinically available dressing material, has been shown to promote acute wound healing through promoting both fibroblast migration (Pizzo et al., 2005) and angiogenesis (Hodde et al., 2001; Hodde et al., 2005), and it can be used as a clinical control to evaluate the efficacy of other dressing materials. However, an animal study showed that the topically administrated ADM did not significantly promote acute wound healing within 1 week post-surgery when compared to the control (Lee et al., 2018). To approach this problem, ADM was administrated through subcutaneous injection, which resulted in significantly higher collagen fiber deposition and microvessel density than the topically administrated ADM (Lee et al., 2018). Whereas, the healing rate appeared not significantly elevated in such an administration method within the initial 7 days of wound healing (Lee et al., 2018). Our study confirmed that ADM was not associated with a significantly higher healing efficacy within the 14-day monitoring span. In contrast, topically administrated Hst1 significantly promoted the healing percentage at as early as day 3 post-surgery. Such a rapid effect of Hst1 may be probably explained by the findings that Hst1 was quickly taken up by cells (Ma et al., 2020) and exert promoting effects on cell spreading and migration within several hours (van Dijk et al., 2017; Ma et al., 2020). Our results also showed that the most effective concentration of Hst1 in this *in vivo* acute wound model was 10 μM, which was consistent with the *in vitro* findings (Shah et al., 2017; van Dijk et al., 2017; Sun et al., 2020). Hst1 promotes wound healing possibly through it potent capacity of enhancing the migration of epithelial cells (Oudhoff et al., 2009a; Oudhoff et al., 2009b; Shah et al., 2017; van Dijk et al., 2017) and fibroblasts (van Dijk et al., 2015), which is mediated by the C-terminal amino acid sequence of Hst1 (Sun et al., 2009) and G-protein coupled receptors (Oudhoff et al., 2009a) and ERK1/2 signaling pathway (Oudhoff et al., 2008).

Considering easy degradation of Hst1, we wished to co-administrate ADM and Hst1 so as to yield a better effect on wound healing since ADM paste might provide a slow release system for Hst1. Furthermore, the different molecular mechanisms of ADM and Hst1 may also contribute to a better effect on wound healing. However, co-administrated ADM and Hst1 did not show a better effect, which did not support our hypothesis. One potential explanation may be that the interaction between positively charged Hst1 with negatively charged ECM components, such as glycosaminoglycans may hinder the release of Hst1, thus reducing its in-site concentration. Furthermore, kallikrein 13 activated by glycosaminoglycans can efficiently cleave peptide substrates at the R-R bond (Andrade et al., 2011), which deactivates Hst1.

Parallel with the migrating process, keratinocyte and epithelial cells also proliferate and differentiate so as to promote thickening and stratifying of the epidermis and to re-establish underlying basal lamina from the margins of the wound inwards. Between 3 and 5 days after injury, collagen production by fibroblasts is deposited and stimulated by TGF-β1, PDGF mainly produced by M2 macrophages (Werner and Grose, 2003). Histomorphometric evaluation of epidermal thickness and collagen deposition can prove the effect of co-administrated ADM and Hst1 on wound healing process (Yang et al., 2018; Liang et al., 2019). In this study, we showed that ADM did not show beneficial effects in either of the two parameters. In contrast, 10 μM Hst1 was associated with significantly higher newly formed epidermal thickness and collagen area than those of control, ADM and ADM + 10 μM Hst1 groups. These results suggested that Hst1 not only efficaciously stimulated cell migration, but also enhanced proliferation and functions of skin cells. Such effects may be attributed to the promoting effects of Hst1 on cell metabolism (Ma et al., 2020) and cell functions (van Dijk et al., 2015; van Dijk et al., 2017) since Hst1 does not promote cell proliferation (Torres et al., 2017).

Angiogenesis to establish a sufficient blood supply provides a favorable microenvironment for epidermal and dermal cell migration and proliferation (Demidova-Rice et al., 2012). The angiogenesis process consists of a series of steps involving various cells such as endothelial cells, inflammatory cells and cytokines such as VEGF, PDGF, TGF-β (Kofler et al., 2005; Naldini and Carraro, 2005). VEGF is a key mediator attributed to endothelial proliferation, migration, tube formation and stimulated the angiogenic cascade (Howdieshell et al., 1998; Carmeliet, 2005). Different ADMs contain different amounts of various angiogenic growth factors, such as VEGF, FGF and TGF-β according to preparation methods (Hoganson et al., 2010), which may contribute to angiogenesis. In this study, the topically administrated ADM did not show beneficial effects on either of the two parameters for angiogenesis: CD31-positive blood vessels and VEGF expression level. Subcutaneously injected ADM is shown to induce more blood vessels than the topical one (Lee et al., 2018). Such an administration method is, whereas, not convenient to apply in clinic and may cause additional pain to patients. In contrast, topically administrated 10 μM Hst1 group presented a higher number of CD31 positive blood vessels and showed a higher VEGF expression level than other groups. Mechanistically, Hst1 directly promotes endothelial cell adhesion, spreading and migration, which is mediated by the activation of the Ras and RIN2/Rab5/Rac1 signaling axis (Torres et al., 2017). Furthermore, Hst1 is also shown to promote angiogenesis and tube formation even in adverse conditions, such as the presence of zoledronic acid (Castro et al., 2019). All these studies indicate that Hst1 is a convenient and potent bioactive agent to promote angiogenesis for accelerating wound healing.

Another paramount biological event for wound healing is inflammatory response, which is essential for hemostasis, attacking invading pathogens and removing tissue debris (Reinke and Sorg, 2012). Within hours after injury, C-reactive protein (CRP) significantly increases, binds to phosphocholine and activate complement system and phagocytosis, thereby clearing necrotic and apoptotic cells and bacteria and contributes. CRP can also increase the secretion and release of IL-1β, IL-6, TNF-α from mononuclear phagocytes (Ballou and Lozanski, 1992), which can stimulate newly attracted monocytes to differentiate into M1 (classically activated and pro-inflammatory) macrophages (Hubner et al., 1996; Werner and Grose, 2003). As early as day 1, infiltrating macrophages in the wound mainly (85%) display an M1 phenotype (The phenotype of murine wound macrophages) (Daley et al., 2010). Macrophages are much larger phagocytic cells that reach peak concentration in a wound at 48 and 72 h after injury. During this period, M1 macrophages transit to M2 macrophages, an alternatively activated, anti-inflammatory and pro-fibrotic phenotype (Hesketh et al., 2017). The proliferation and migration of M2 macrophages secret TGF-β1, VEGF-α contributed to the proliferation and migration of fibroblasts, endothelial cells, epithelial cells, angiogenesis and re-epithelialization (Goren et al., 2009). The dominating macrophage population in the wound at day 5 is the M2 macrophage population (Lucas et al., 2010). However, a prolonged inflammation may enhance risks of delayed healing and excessive scarring. Our study showed that 10 μM Hst1 group presented a lower number of CD68-positive macrophages and showed a lower IL-1β, IL-6, TNF-α and CRP expression level than other groups at day 7 post-surgery. To our knowledge, this was the first study to show that Hst1 could significantly down-regulate inflammation in wound healing. The underlying mechanism for this function of Hst1 still remains to be elucidated.

In this study, we showed that ADM appears not to be an ideal carrier of Hst1. Further efforts should also be taken to look for a promising wound dressing material to provide a sustained release system for Hst1. Recent studies show that conductive photothermal self-healing nanocomposite hydrogels wound dressing bear good biocompatibility, biodegradability, mechanical properties, photothermal properties, antibacterial and sustained drug release behavior for wound healing application, even in bacteria-infected wounds (Liang et al., 2019). It would be worth evaluating the efficacy of Hst1 in combination with such multifunctional hydrogels (He et al., 2020; Liang et al., 2020). One limitation of skin model in mice is that contracture also plays a role in wound healing. A skin wound healing model in pig that is more similar to human skin and bear much less interference from contracture (Sullivan et al., 2001), would be necessary to further corroborate the application potential of Hst1 in clinic.

In conclusion, topical administrated Hst1 at 10 μM could significantly accelerate wound healing by promoting angiogenesis, re-epithelialization, and collagen production as well as suppressing inflammation, which indicated a promising application in managing acute wound healing.

## Data Availability Statement

All datasets presented in this study are included in the article/supplementary material.

## Ethics Statement

The animal study was reviewed and approved by the Animal Care Committee of General Hospital of Southern Theater Command. The animal ethics approval number was 2019051701.

## Author Contributions

BC, GW, TF, FB, and XL contributed to conception and design of the study. XL, LC, HL, MP, ZY, and CC performed the study. XL, LC, and HL organized the database. XL, GW, and LC carried out the data analysis. XL and GW wrote the manuscript. All authors contributed to manuscript revision, read and approved the submitted version.

## Conflict of Interest

The authors declare that the research was conducted in the absence of any commercial or financial relationships that could be construed as a potential conflict of interest.
